# Excessive bleeding is a normal cleansing process: a qualitative study of postpartum haemorrhage among rural Uganda women

**DOI:** 10.1186/s12884-016-1014-9

**Published:** 2016-08-08

**Authors:** Sam Ononge, Elialilia Sarikiaeli Okello, Florence Mirembe

**Affiliations:** 1 Department of Obstetrics and Gynaecology, College of Health Sciences, Makerere University, Kampala, Uganda; 2 Department of Psychiatry, College of Health Sciences, Makerere University, Kampala, Uganda

**Keywords:** Postpartum haemorrhage, Home births, Intra-partum practices, Delay seeking care

## Abstract

**Background:**

Postpartum haemorrhage (PPH) remains the leading cause of maternal morbidity and mortality worldwide. The main strategy for preventing PPH is the use of uterotonic drugs given prophylactically by skilled health workers. However, in settings where many women still deliver at home without skilled attendants, uterotonics are often inaccessible. In such cases, women and their caregivers need to recognize PPH promptly so, as to seek expert care. For this reason, it is important to understand how women and their caregivers recognize PPH, as well as the actions they undertake to prevent and treat PPH in home births. Such knowledge can also inform programs aiming to make uterotonics accessible at the community level.

**Methods:**

Between April and June 2012, a phenomenological study was carried out in a rural Ugandan district involving 15 in-depth interviews. Respondents were purposively sampled and included six women who had delivered at home in the past year and nine traditional birth attendants (TBAs). The interviews explored how PPH was recognized, its perceived causes, and the practices that respondents used in order to prevent or treat it. Phenomenological descriptive methodology was used to analyse the data.

**Results:**

Bleeding after childbirth was considered to be a normal cleansing process, which if stopped or inhibited would lead to negative health consequences to the mother. Respondents used a range of criteria to recognize PPH: rate of blood flow, amount of blood (equivalent to two clenched fists), fainting, feeling thirsty, collapsing or losing consciousness immediately after birth. As a group, respondents seemed to correctly identify women at risk of PPH (those with twin pregnancies, high parity or prolonged labour), but many individuals did not know all the reasons. Respondents used cold drink, uterine massage and traditional medicine to treat PPH.

**Conclusion:**

The community viewed bleeding after childbirth as a normal process and their methods of determining excessive bleeding are imprecise and varied. This opens the door for intervention for reducing delays in the home diagnosis of PPH. This includes increasing awareness among TBAs, women and their families about the risk of death due to excessive bleeding in the immediate postpartum period.

## Background

Labour, delivery, and the first 24 h after childbirth (the immediate postpartum period) has been found to have the highest risk of maternal deaths, accounting for approximately 61 % of deaths [1]. Many of these are due to postpartum haemorrhage (PPH) which, at 27.2 % of obstetric deaths, is the commonest single cause of death [2]. Like many sub-Saharan African countries, Uganda experiences high maternal mortality and low skilled attendance at birth. The 2011 Uganda Demographic Health Survey reported a ratio of 438 maternal deaths to 100,000 live births, of which approximately 25 % were due to PPH [3]. In addition to death, PPH leads to anaemia and its consequences, (including failure of lactation and a consequent inability to feed the newborn) [4, 5], and increased susceptibility to postnatal infection [6].

Using risk factors to predict which women will develop PPH is difficult because two-thirds of those who develop PPH have no measurable risk factors [7]. For this reason, all women are considered to be at risk, and are advised to prepare for a facility birth with a skilled attendant. Moreover, women are advised to make preparations for birth and potential complications through “birth preparedness” and “complication readiness”.

According to Thaddeus and Maine, the maternal mortality can be ascribed to three delays [8]. The Three Delay Model of Safe Motherhood programs provides a useful framework for explaining the relevant context for the life-threatening obstetric complications like PPH. The model proposes that delays in obtaining appropriate emergency obstetric care occurs at three points; 1. recognition of a problem and decision to seek health care, 2. reaching health care facilities, and 3. receiving emergency obstetric care once at the health facility [9]. It is proposed that each delay needs to be addressed, and that a reduction in the delays will lead to decrease in maternal mortality and morbidity. Timely recognition and knowledge of the signs of PPH is therefore a critical step in seeking to reduce PPH deaths. In settings with many home births, women or their attendants may view excessive bleeding at childbirth as normal because of cultural perceptions related to bleeding [10]. If excessive bleeding is perceived as normal, it may cause the woman or her attendant to wait to see if the bleeding will stop or subside on its own. Thus, the perceptions of *normal* bleeding may be an important factor in delaying the decision to seeking health care. Studies documenting communities’ recognition of PPH in home births suggest it is poor [11]. For example, a study in India documented that of the women who experience life-threatening bleeding after childbirth, only 23 % were perceived to have excessive bleeding by the women themselves or their birth attendants [11], and the poor recognition of PPH influenced their decisions to seek health care. A study in Malawi, exploring factors that contribute to the high magnitude of PPH in the hospital found the lack of timely referral from traditional birth attendants (TBAs) to be an important factor [12].

In Uganda, although the Ministry of Health policy recommends that childbirth should occur under supervision of skilled attendants, up to 42 % of the women deliver outside a health facility [13]. In these home births, women are assisted by relatives or TBAs, or they may deliver on their own. But there is little documented about lay perceptions of postpartum bleeding in Uganda. The aim of this study was to gain a deeper understanding of the women’s and TBA’s experiences and perceptions of excessive bleeding after childbirth, and to explore how they identified when bleeding was excessive. The study also set out to explore practices when women experienced excessive bleeding. In this way, we sought to highlight the challenge associated with PPH recognition and to use the information obtained to improve public health education messages. In addition, understanding lay perspectives will also help inform and develop interventions (community misoprostol distribution), programs and policies that will assist women who give birth without skilled attendant.

## Methods

### Setting

This study was carried out between April to June 2012 in Mpigi, a rural district in central Uganda. Mpigi District is located 33 km from south west of capital City Kampala, and is composed of six Sub-Counties and an Urban Town Council. It has 56 parishes and 335 villages. The main economic activities in Mpigi District are subsistence farming and fishing. The population is of relatively homogenous ethnicity, with a majority belonging to the Baganda ethnicity. Mpigi District’s health infrastructure follows the Uganda National Health System, and is divided into several levels from Health Centre I to IV. Health Centre Is are at village level and cover a population of around 500 people. They are served by village health teams (VHTs). These are made up of community health workers selected by village members, and they facilitate health promotion, community participation and utilization of health services. Health Centre IIs are at the parish level and offer outpatient services to a population of around 5000 people. The standard staffing for a Health Centre II is an enrolled nurse, a midwife and a nursing assistant. Health Centre IIIs, serve a Sub-County with an estimated catchment of 25,000 people [14], and are staffed by clinical officers, nurses, midwives, laboratory technician, and a health inspector. Mpigi District has 13 Health Centre IIIs and maternity services offered at this level, which include basic emergency obstetric care. Mpigi District has six Sub-Counties. Health Centre IVs serve a County and according to Uganda National Health System, they serve an estimated catchment population of 95,000 people. Health Centre IVs are staffed as for Health Centre IIIs, but with the addition of a medical officer(s) and an anaesthetic officer. They provide comprehensive emergency obstetric care, including caesarean section. Though Mpigi District has two Counties, it has only one Health Centre IV, with plans to upgrade it to a district hospital. The district also has a private, not-for-profit hospital (Nkozi) which offers medical services to those who can afford the fees.

The health indicators and the utilization of health facility services of Mpigi District are comparable to the national figures: approximately 95 % of the pregnant women attended antenatal clinic at least once, but only 58 % deliver with a skilled attendant [15].

### Study design

To obtain a deeper understanding of the women’s and TBAs’ experience of bleeding after childbirth, a phenomenological approach was adopted [16]. Phenomenology is concerned with the lived experience of the people involved. In this case, it was with the birth, seeking care, patterns of meaning related to bleeding after childbirth. It was used because the distinction between normal and excessive (serious) bleeding is poorly understood in many communities [10] and the design enabled us to gain insight into individuals’ experiences and into the meanings they attach to these experiences.

### Sampling procedure and data collection

The study recruited informants from Kamengo, Buwama and Muduma Sub-Counties in Mpigi District. We sought women who had given birth at home within the previous year, and trained or untrained TBAs who had assisted at least one woman give birth at home within the previous 6 months. The informants were purposively sampled from the study villages with the help of the local Sub-County VHT coordinators. The sampling aimed to ensure that women with varied knowledge of childbirth and excessive bleeding were included in the study. A formal request was made to respondents for them to participate in the interviews, and none declined. The interviews were conducted in the participant’s home and lasted for 45 to 60 min on average. They were conducted by a female social scientist, trained in anthropological research, and supported by a note taker. Both were fluent in Luganda, the local dialect language. Before each interview, the researchers clarified that they had no involvement in district administration, that the participant’s particulars would remain confidential, that their participation was voluntary, and that responses would not lead to any retribution. Individual verbal consent was sought from all the informants and permission to use the tape recorder was requested. The interviews started with open-ended question: “can you tell me about the recent birth(s) you had or conducted?” To obtain a deeper understanding of the participants’ experience with excessive bleeding, they were subsequently asked: “Can you tell me about blood loss after childbirth? How do you tell that bleeding is excessive?” Clarifying questions were asked when needed. We also asked what actions informants took when they recognized that bleeding was excessive. The interviews also explored perceptions of the risk factors for PPH and the reasons that women opted for a home birth. The tape-recorded interviews were immediately transcribed and translated to English by the interviewer for non-native speakers and for analysis.

### Analysis

Phenomenological descriptive methods were used to analyse the data [16]. Transcripts were read several times to gain an understanding of the text. During the reading, the overall meaning was identified through differences and similarities recognized through the text. The text was then divided into small parts, called ‘meaning units.’ Meaning units were then classified into different clusters and through the different clusters and the text as a whole, the essential structure of the studied phenomenon emerged. In this part of the analysis, we continually moved from the whole text to the parts, and back. The first author and another researcher performed the analysis manually. The meaning units were discussed with co-authors.

### Ethical approval

Ethical approval was obtained from the ethics and research committees of School of Medicine Makerere University College of Health Sciences Committee and the Uganda National Council for Science and Technology. Verbal consent for participation and audio recording of the discussion was obtained from each participant.

## Results

A total of 15 interviews were conducted, six with women who had delivered and nine with TBAs. As shown in Table 1, the age range of the participants was from 20 to 80 years. Almost all the TBAs were grand-multiparous women and had received some training from World Vision or the District Health Office.

**Table 1:** Demographic and health characteristics of the study participants

Participants			
Traditional Birth attendant	Age in years	Parity	Years of experience and training
TBA 1, Buwama	80 years	Para 10.	More than 60 years of practice. Learnt from her mother. Untrained. All her children were delivered at home.
TBA 2, Kamengo	65 years	Para 10.	Thirty years of practice. Learnt from her mother in law. Trained by World Vision
TBA 3, Kamengo	56 years	Nulliparous.	Thirty-two years of practice. Learnt from her mother. Trained by World Vision.
TBA 4, Buwama	52 years	Para 5.	Twenty years of practice. Learnt from her mother. Trained by World Vision.
TBA 5, Buwama	46 years	Para 12.	Thirteen years of practice. Learnt from her aunt. Trained by World Vision.
TBA 6, Kamengo	60 years	Para 7.	Forty years of practice. Learnt from her aunt. Trained by World Vision.
TBA 7, Muduma	70 years	Para 10.	Fifteen years of practice. Worked initially as a nursing aide at the hospital; started assisting women to deliver at home upon retirement.
TBA 8, Muduma	42 years	Para 10.	Sixteen years of practice. Learnt from her mother. Trained by the District Health Office.
TBA 9, Kamengo	67 years	Para 6.	Eighteen years of practice Training by World Vision.
Women		Parity	Delivery history
Woman 1, Muduma	20 years	Para 1.	Home birth
Woman 2, Muduma	22 years	Para 3.	Last delivery was the only one at home
Woman 3, Kamengo	27 years	Para 4.	Last two deliveries occurred at home
Woman 4, Kamengo	32 years	Para 8.	Seven deliveries were at home and one at the TBA’s home.
Woman 5, Buwama	47 years	Para 9.	All nine deliveries at home. Four were delivered alone.
Woman 6, Buwama	28 years	Para 2.	Both deliveries at the road side

The women and the TBAs described what occurs at childbirth in relation to bleeding using the following categories: they suggested that bleeding after childbirth is normal and serves as a cleansing process. They described fears that blood clots retained in the womb might result in several complications such as pains after birth, infection, and blockage of the fallopian tubes. Respondents expressed the meaning of excessive bleeding in various ways. For instance, excessive bleeding was expressed in terms of a rapid rate of blood flow; a large amount (the equivalence of two clenched fists); and signs of hypovolemic shock (fainting, collapsing or losing consciousness). They acknowledged there was no remedy for treatment of excessive bleeding in home births. Respondents correctly described the risk factors for excessive bleeding as twin pregnancies, high parity births, and prolonged labour. They also ascribed excessive bleeding to contraceptives use.

### Defining excessive bleeding

The respondents had several ways of defining excessive vaginal bleeding after childbirth. Most TBAs used their experience assisting many women to tell that a woman has lost more blood than usual. However, the TBAs acknowledged that excessive bleeding was rare event and many reported not having witnessed a case of excessive bleeding in the previous year.

To tell that bleeding is excessive, respondents often spoke of assessing the rate or speed of blood flow, having to change pads with a high frequency or of the expulsion of clots. However, a first time mother who did not have prior experience of childbirth might not be able to rely on these methods. A 20 year old, woman noted her lack of knowledge in telling whether bleeding was excessive, and said she would have been unable to tell unless somebody else told her she had lost a lot of blood. Blood loss “equivalent to two hand fists” was a method TBAs also used to define PPH. Some TBAs had learnt this from trainings organised by a non-governmental organization, World Vision. World Vision had conducted trainings in the area approximately 10 years before the study. However, a 56-year-old TBA from Kamengo reported a more specific measurement of blood loss equivalent to 500 mls of blood. She often used a plastic mug to measure the blood after delivery of the baby and placenta. According to her, blood loss more than the plastic mug was a sign that the woman has PPH.

*“I have a plastic mug that I use when a mother delivers. I collect blood from the mackintosh into this mug and that helps me estimate how much the woman has lost. If a woman I have delivered bleeds more than one cup, then that is excessive bleeding, I refer her to hospital for further management”* (TBA 3).

In the same sub-County (Kamengo) another TBA also trained by World Vision reported a threshold blood loss of 1000 mls or more for PPH diagnosis.

*“We were told from the training organized by World Vision that the right amount to be lost after delivery should be not more than two mugs. When blood loss is more than two mugs, then the mother is in danger”* (TBA 9).

From the data, it also emerged that the signs and symptoms of shock were another method used to recognize PPH. Women referred the complaints of feeling dizzy, fainting, sweating, or collapsing as the symptoms excessive bleeding. They reported that when any of the above symptoms were experienced immediately after childbirth, the most likely cause was excessive bleeding. A TBA and a woman described the symptoms and signs that alerted her as follows:

*“When a woman after delivery complains of dizziness, starts sweating and losses consciousness, she is in danger. She fails to sit up on the bed or she is feeling palpitations, that woman has excessive bleeding”* (TBA 6).

*“After delivery, little blood was coming as usual, but within a short time, I realized that the cloth I had used as a pad was soaked with blood. Besides that, I was also feeling dizzy and I was not understanding things around me until I was given cold water to drink”* (Woman 5). A mother of four children gave birth to a baby alone in her house and she managed to deliver the placenta. She described PPH as that vaginal bleeding which is more than the amount lost in menstruation period:

*“In the morning after giving birth, I compressed my tummy with warm banana leaves, then blood flow increased. I removed the cloth that I used as a pad and replaced it with another. Within short time that pad was also soaked. This flow was more than my menstrual period bleeding.”*(Woman 3)

#### Vaginal bleeding after childbirth as a normal and cleansing process

The respondents stated that bleeding after childbirth was normal and that every woman was expected to lose blood. They believed that the bleeding after childbirth would stop on its own and should not be inhibited. Postpartum bleeding, like menstruation, was considered to be a natural, cleansing process. There was a belief that some blood accumulated during the 9 months of pregnancy and that it should be allowed to flow out during and after childbirth. Inhibiting bleeding would mean that the some “dirty” or “bad” blood would remain in the woman’s womb (uterus) and mostly result in complications. Allowing some blood flow was believed to help restore the woman’s health and cleanliness. A TBA described how bleeding after childbirth makes the postpartum woman clean:

*“When that blood that flows immediately after the baby is born comes out, you are now clean”*. (TBA 8)

The 20 year old delivered her first baby at home with the help of an aunt and described what her aunt did to expel the “dirty blood”, and why it was necessary:

*“I gave birth to my baby in the middle of the night. In the morning, my aunt got some banana leaves with warm water and compressed my abdomen to expel the bad blood. She repeated the process in the evening. … The blood that flows after childbirth is bad blood because for the whole pregnancy you are not menstruating and this is a long period. When that blood remains in your womb, you might get some complications”.* (Woman 1)

#### Fear of blood retention

Since bleeding was considered as a cleansing process, women believed that when blood was stopped or prevented from coming out, it would accumulate in the womb. The retained blood was then associated with causing harm, such as infection or sepsis. Respondents believed that the retained blood could cause the womb to rot and thereby result in death. Some respondents believed that ‘bad blood’ blocked the fallopian tubes and prevented woman from having more children in the future. They also believed that retained blood caused after birth pains, and prevented these by allowing blood to flow. To facilitate blood flowing out and remove the retained blood, every woman was expected to undergo “abdominal compression” after childbirth using a warmed towel or boiled banana leaves, 6–12 h after delivery. Respondents reported knowing the retained blood was out when blood clots were expelled or when they observed a slight increase in bleeding compared to before the abdominal compression. Abdominal compression was also thought to ensure a quicker recovery from the strain of labour, with the ultimate aim of returning to daily chores. Some respondents also administered traditional medicine that they believed helped to clean the uterus.

*“The dirty blood may retain in the womb and it is difficult to treat. When you do not press the abdomen, you will get complications, which may call for an operation, because you did not take good care of yourself. Even if they give you tablets to swallow at the hospital, you have to press your tummy. Traditional medicine (Kamunye) is also very good in cleansing the womb, however, you must use it after pressing your tummy.”* (Woman 2)

A TBA with over 30 years of experience assisting women to deliver acknowledged having delivered a woman who had very little vaginal blood loss after childbirth and delivery of the placenta. The TBA’s concern was that the bleeding was very little, and her immediate reaction was to refer the woman to the hospital because she had a problem. The TBA described her experience with the woman:

*“I got a woman who didn’t bleed a lot after delivery. My concern was that the blood loss in vagina was very little. This was not normal from what I am used to seeing when I assist women. I told her to go to the hospital, but she refused because she was not feeling bad.” (TBA 2)*

Because of notion of referring postpartum blood as ‘bad or dirty’, women may resist any medication that stops or reduces blood flow. One of the respondents was against any medication that would reduce bleeding after childbirth. She would only swallow the tablet to reduce excessive bleeding if persuaded by her birth attendant.

“*Personally I can’t swallow those tablets that stop bleeding because I want the blood to come out. That blood is bad and dirty, so it must come out. …If my attendant gives me those tablets, I will pretend that I have ingested them and keep them. I will only ingest them to make her happy if she insists that I swallow when she is observing me.”*(Woman 1)

### Perceived risk factors for PPH

The respondents expressed difficulty in predicting which women would be more likely to experience PPH, although they were aware of some risk factors. They attributed excessive bleeding to having given birth to more than five children, which according to them made the uterus “tired”. Other risk factors mentioned were retained placenta, bleeding before childbirth, prolonged labour, twin pregnancies and anaemia in pregnancy. They also reported that women pregnant for the first time were more likely to bleed because of injuries to their birth canal. Any pregnant woman who presented to the TBAs with any of above factors was considered to be at high risk, and TBAs said they referred them to the hospital. Interestingly, none of the respondents mentioned failure of the uterus to contract (uterine atony) after childbirth as a cause of excessive bleeding, but indirectly mentioned some causes of uterine atony, including grand-multiparity, twin pregnancy, retained placenta and prolonged labour.

The use of modern contraceptive methods were mentioned as a risk factor of excessive bleeding. Respondents perceived that contraceptive methods that caused amenorrhea (stopped women from menstruating) could lead to excessive bleeding after childbirth, presumably because this blood had accumulated prior to the pregnancy. Some also mentioned that aside effect of other contraceptive methods was to prolong menstrual bleeding in non-pregnant women so, by analogy, a woman who used contraceptives prior to giving birth was more likely to get a PPH.

*“Women who have been on family planning methods bleed a lot after childbirth. These women will have taken some time without menstruating, for example three years and immediately she gets pregnant. When she is giving birth, she will bleed a lot.”* (TBA 5).

#### Actions women take in response to excessive bleeding in home birth

The majority of the women reported knowing no home remedy to stop excessive bleeding at childbirth. However, women knew that the health facilities had injections that were given to reduce excessive bleeding after childbirth.

*“At the hospital, the midwife gave me an injection and she told me that the injection was to prevent excessive bleeding. When I delivered at home, I did not receive any medicine to reduce excessive bleeding. I don’t know what other women who deliver at home use to prevent bleeding.”* (Woman 2)

The TBAs reported that women, who experienced excessive bleeding after home births, were referred to the health facility. Interestingly, three TBAs kept the drug ergometrine at their homes, which they could administer to the woman after childbirth. On a few occasions, some TBAs reported how they telephoned for help from a health worker in nearby health facility. They reported good working relationships with formal sector health workers s as two of the TBAs explained:

*“If excessive bleeding happens, I massage the abdomen and I get out all the ‘dirty’ blood because it also leads to PPH. I have an injection that I administer to the mother so that the bleeding reduces. If a mother tells me that she usually bleeds a lot after every delivery, I do not wait any minute. I give the mother the injection immediately after the baby is born.”* (TBA 7)

*“Normally I call the health worker and she comes. She gives an injection and the bleeding stops.”* (TBA 6)

Many TBAs reported using cold water or soda to stop excessive bleeding. They were convinced that this prevented and treated PPH. They claimed this practice has been there for a long time and that they have seen it work. They believed that a warm drink would increase blood flow in the body and simultaneously increase vaginal bleeding.

*“If the blood loss is much, I can give the mother cold water or any drink that is cold, then the flow reduces. If you don’t do that, she might lose all the blood in the body.”* (TBA 8) *“Apart from cold water, I don’t give anything else. I don’t even give any herbs to stop bleeding. When you give cold water, the bleeding is controlled. When I give the woman the water to drink and it doesn’t stop, then I refer her to the Health Centre so that she gets an injection which will control the bleeding.”* (TBA 5)

Contrary to the belief held by many TBAs and women on effectiveness of cold water, one 70 year TBA from Muduma did not believe that cold drinks reduced excessive bleeding. Her background experience as a nursing assistant at the hospital could have informed her actions. She questioned the rationale of a cold drink and stressed that it may do more harm than good.

*“Cold water can make matters worse. I can not use it all. I give warm tea but I don’t give cold water to the mother. How can you give cold water to a woman who has delivered? The mother needs something warm to go in the stomach.”* (TBA 7)

The majority of TBAs reported that they no longer used traditional medicine to reduce or stop excessive bleeding. However, this followed the training coordinated by the District Health Office and World Vision where the use of traditional medicine was discouraged and sometimes criminalised. Nonetheless, some TBAs still gave traditional medicine when they observed excessive bleeding or when the placenta was retained.

*“When I was giving birth, my mother in law used to give me a certain ‘mumbwa’. Ever since we went for these trainings, we were told not to give any herbs to the mother.”* (TBA 2)

Mumbwa (sometimes referred as Emumbwa) are clay rods containing one or more local herbs. Women who used them were seldom aware of the specific herbs within these clay rods. The multiple herb-containing clay rods were taken orally, and were assumed to serve as wellbeing supplement for the foetus and mother [17].

### Why do women not deliver at the health facility?

The respondents mentioned benefits of giving birth at the health facility and being cared for by a trained health worker. However, they also mentioned problems like; lack of birth preparedness, lack of transport, delivering at night and fear of mis-treatment at the hands of health workers. The respondents cited these factors as barriers to delivering at the health facility. They said that giving birth at home was cheaper than in a health facility. The latter involved transport costs, paying for the delivery and purchase of particular items to be used at hospital.

*“Sometimes I hear other women say that there is no money, let me go to the TBA. The TBA will get her money in installments, which cannot be the case when you go to the hospital.”* (Woman 4*“There are times when the woman does not have the necessary things needed in the hospital. The nurses will ask for gloves, cotton, clothes for baby etc., and yet she does not have them. So, when she comes here, I devise a means of helping her. I also give her water to drink and food to eat.”* (TBA 2)

Sometimes, labour started at night and transport to the health facility at that time was a challenge. In other cases, labour progressed quickly and there was no time to get to the health facility. A mother of two described how transportation difficulties prevented her from giving birth at the hospital.

*“I have had the two births at the road side. I prepared to leave the house but I did not reach the hospital. In the recent birth, I got the labour pains at night and we went to the road to catch a Boda boda (motorbike) to take me hospital in Buwama. We failed to get any transport at that time. I could not sit on a bicycle, the only transport my husband had. When my husband saw that the labour pains were frequent and there was no Boda boda to take me, he rode his bicycle to Buwama to bring a motorbike to take me to the hospital. When I saw that I could not contain the pains, I slowly walked and reached a home near the road, where I found a woman who helped me give birth. By the time my husband arrived with motor bike, I was pushing the baby.”* (Woman 6

Previous negative experiences at the hospital were other reasons women preferred to deliver at home. The poor attitude of the health workers discouraged women from wanting to give birth at the health facilities.

*“The nurses are very rude to the mothers in labour. So you make a decision to stay at home.”* (Woman 2)

*“There are times when you go to hospital to deliver and the nurse asks irritating questions like; how many children do you have? Are you looking for the president? Such comments make women not want to deliver at the health centre. A nurse can even call her colleagues to come and see a mother who has ten children, and is pregnant again. This will make the mother not to deliver from the hospital.* (TBA 2)

## Discussion

The principal conclusions of this study were that; bleeding after childbirth was perceived to be cleansing process, that diagnosis of PPH was often delayed, that the community recognized there were few treatment options for PPH and those they did were unlikely to have a physiological basis.

There was a strong belief amongst both individual women and TBAs that blood after childbirth was “dirty blood”, and that it was normal for every woman to bleed to become clean. The community described bleeding after childbirth as a cleansing process that is good for the body, and that stopping the bleeding may cause “the dirty things” to stay in the body. The respondents related childbirth bleeding to menstrual bleeding which many societies consider to be a monthly cleansing process [18]. Many women get concerned when they do not have monthly blood flow. They are worried that this blood, which was termed “dirty,” accumulates in the womb and is likely to cause problems. The women in this study believed that the blood after childbirth was menstrual blood that had accumulated in the uterus for the nine months when they were pregnant and must be allowed to drain out. Whilst menstrual loss may not have immediate implication to the woman’s health and is usually self-limiting, the postpartum bleeding is usually more substantial because of the physiological changes of pregnancy and the placental site. The underlying belief that blood of childbirth is dirty has been reported among women in Morocco who also believe that blood of childbirth is bad and potentially poisonous inside the body [19]. In Bangladesh, a belief that bleeding is a cleansing process is also held by some skilled birth attendants [20]. When women and TBAs believe that bleeding of childbirth is a cleansing process, it is likely that they may delay seeking care because the blood loss is viewed as normal. As reported elsewhere, the perception that blood is dirty may also result in limited contact between the mother and other family members [10]. This may also contribute to delay in consultation by the woman or in seeking skilled care, even if it is threatening the woman’s life.

Conversely, too little bleeding was perceived as retention of bad blood in the uterus and was associated with complications. Respondents thought that retained clotted blood in the womb caused abdominal pain (after birth pains), delayed recovery processes, and caused postpartum haemorrhage and puerperal infection. To prevent retention of clotted blood in the womb, the women and TBAs have traditional medicine that was ingested after birth for the next 24 h. In addition, they routinely practiced abdominal compression to expel the uterine contents. This practice of abdominal compression has been observed in Bangladesh [21] and also reported by Obermeyer in Morocco [19]. The communities in Matlab in Bangladesh had traditional medicine they believe breaks down the blood clots in the womb [21]. Meanwhile, women in Nepal would encourage the mother to do hard work to accelerate bleeding. While it is understood that after pains is a biological phenomenon that controlling excessive bleeding, the person’s fear of clotted blood in the womb and practice of abdominal compression may induce fresh bleeding. This has clinical implications in that this practice may cause postpartum haemorrhage. There is a need to educate communities about harmful practices that may increase postpartum morbidity and mortality. Education of communities can be through antenatal clinic, mass media and VHTs.

### When does bleeding become excessive?

From the study, the participants had varied ways to define excessive bleeding. The frequency of changing sanitary pads, and the forceful nature or speed of blood flow were used to tell that the bleeding is excessive. Although these are subjective, they are recognized to be useful signs to raise concern about excessive blood loss. However, in a community where bleeding was considered as a cleansing process, and the lack of bleeding as a problem, it may be difficult for a woman or her attendant to distinguish between bleeding that “cleanses” the uterus and that which is dangerous. In addition, the use of different types of materials as sanitary pads to absorb postpartum blood may confound recognition of excessive bleeding. Some women use cotton, while others have pieces of clothes as sanitary pads. These materials have different blood absorption capacities. This may suggest that recognition of excessive bleeding may be delayed depending on material used. In addition, the TBAs use of symptoms like dizziness, sweating, thirsty or fainting, are important signs of hypovolaemia when there is no objective method. However, these symptoms only occur as late symptoms of PPH, and are not specific to excessive blood loss. The physiological adaptation during pregnancy provides a compensatory reserve such that healthy women can stand acute blood loss at delivery of up to 1000 mls [22]. When women bleed in excess of this after childbirth, they can experience dizziness, sweating, thirsty and fainting, all symptoms of hypovolemic shock [23]. In healthy pregnant women, the masking of signs and symptoms of shock hinder recognition of excessive bleeding and ultimately delays seeking immediate care. It is also recognized that severe bleeding frequently stops on its own [24], and this leads the women or the birth assistants to delay seeking care. Most of the TBAs reported that the problem of excessive bleeding was rare in their setting. Therefore, relying on the symptoms of shock to diagnose excessive bleeding may lead to under-reporting of excessive bleeding. Meanwhile, the TBAs who had received some training on safe delivery reported a more objective method of blood assessment: any blood loss more than two hand fists was considered as excessive bleeding, where one fist was equivalent to 300 mls. Basing evaluations on a hand fist in itself, is not precise. Due to the imprecise nature of methods of recognition of excessive bleeding after delivery, communities need to know that it is normal for every woman to have some bleeding after childbirth. However, when the blood loss is half a litre or more, the bleeding is heavy and immediate action is necessary. For example, actions like seeking help, emptying the bladder, rubbing the uterus or breastfeeding the baby can be communicated to the communities through antenatal clinics, mass media or VHTs. In addition, these simple actions can be delivered through mobile phone text messages (smart messaging service) in local languages in the region as most Ugandans have mobile phones [13].

### Risk factors for excessive bleeding

While the TBAs beliefs regarding risk factors for PPH differed from biomedical concepts of the pathophysiology of excessive bleeding, there were some similarities. For instance, the dangers associated with too many births, retained placenta, prolonged labour and twin pregnancies are documented biomedical risk factors for PPH [25]. The TBAs’ knowledge of risk factors could have been influenced by the training that most TBAs had received. Surprisingly, failure of the respondents to mention uterine relaxation (atony) after childbirth as a cause of excessive bleeding could suggest a lack of knowledge on the leading cause of PPH [26]. However, the belief associating family planning methods with excessive bleeding after childbirth is not consistent with the precepts of biomedicine. This belief may constitute an obstacle to the use of modern contraceptives.

### Actions to manage excessive bleeding

There was an expression by the participants that there were no effective methods available at the community level to handle excessive bleeding. Some of the TBAs were not sure if the herbs they gave to women were stopping excessive bleeding. However, for those TBAs who did use herbs to treat PPH, reliance on them may be an obstacle to seeking health care. The TBAs (especially those who had been trained) knew that health facilities had better medication to stop bleeding after childbirth. When a woman under their care experienced excessive bleeding, they reported they would refer her to the health centre. Some of the TBAs had adopted practices that were performed by health workers at the health facilities for treatment of excessive bleeding after childbirth. For instance, they learnt how to give injections to stop excessive bleeding and some stocked injectable medicines, ergometrine, in their homes. In addition, some of the TBAs had a good working relationship with the health workers from the nearest health centre. They would ask the nurse to come and give the injection.

From the biomedical perceptive, the use of cold water to treat excessive bleeding after childbirth could result in deterioration in the physical health of the woman [27]. Some women may be in hypovolemic shock and cold drinks may worsen the hypothermia already experienced. Giving oral drinks does little to replace the blood volume being lost and reliance on it may delay the seeking of health care. To reduce morbidity and mortality secondary to excessive bleeding, we need to educate communities on more effective methods to treat PPH. The importance of delivery with skilled birth attendant should be emphasized and promoted in educational messages to the women, household members and TBAs. In addition, the use of misoprostol for prevention and treatment of PPH should be discussed with the women and household members present during home births. Misoprostol has been shown to be safe and effective in settings where the administration of injectable uterotonics is not feasible [28–31]. As Robinson et al commented in their paper, education of women and household members on how and when to swallow the misoprostol is one avenue to increase use of misoprostol at the time of home delivery [31]. The education can be done by VHTs, and where they have been used as community educators in misoprostol community, acceptability of misoprostol is enhanced [31, 32]. However, governments should train and support VHTs as well as TBAs on use of misoprostol for prevention and treatment of PPH.

### Preference for home births

A factor that has been found in many developing countries to discourage women from having their deliveries at the health facilities is the poor attitude of some health workers [8, 33]. Previous negative experience at the health facility may influence the choice of home birth, and may contribute to delay in seeking health care. As in previous studies [33, 34], we found that the onset of labour at night, and precipitate labour in some women prevented them reaching the health facility. Getting to the health facility at night was a challenge when communities depended on motor bikes as a means of transport. In some areas the motor bikes riders do not move at night because of insecurity. Women living far from a health facility, whose onset of labour pains started at night, or have precipitate labour, most often have home births as their ultimate choice.

As reported in other studies, we found that lack of money to pay for transport and cost of care at the health facility was a hindrance to seeking delivery services at the health facility [8, 35–37]. Poor women without money are more likely to stay away from the health facility for fear of being embarrassed by health workers when asked for items such as cotton and gloves, which are required at childbirth. Consequently, poor women rely on the services of TBAs who are seen to play important role during childbirth, and are considered cheaper than going to a health facility [38]. TBAs often have a flexible mode of payment for their services, making money less of a hindrance to accessing care from them. In addition, TBAs psychological support and provision of food during labour makes their services attractive to the women.

### Study limitations

We acknowledge that there are methodological limitations that may impact on our findings. The first limitation of our study is on the sample of participants. Study participants were drawn from one ethnic group, the Baganda. Our findings may therefore not be generalized to other settings with different cultural practices and beliefs regarding child bearing. Secondly, the presence of the principal investigator (an obstetrician), during most of the interviews could have influenced our findings, although using an independent social scientist moderated the in-depth interviews minimized this bias. Thirdly, neglecting the inclusion of women who delivered at the hands of skilled birth attendants restricted our focus to the experiences of women who delivered at home or with help of TBAs.

## Conclusion

Women and TBAs viewed bleeding after childbirth as a normal process and their methods of determining excessive bleeding are imprecise and varied. These factors may markedly delay the diagnosis of postpartum haemorrhage. To reduce the delays in the decision to seek care, there is a need to raise awareness among TBAs, communities, women, and their families about the risk of death in the immediate postpartum period due to excessive bleeding. The women need to know that bleeding after childbirth is dangerous and not desirable. In addition to creating awareness, the authors support strategies involving VHTs and TBAs in the prevention of PPH. Strategies of training TBAs on the correct dosing and timing of misoprostol administration for PPH prophylaxis should be encouraged. This could increase the number of women accessing a uterotonic (misoprostol) at birth for prevention of PPH. However, strategies to encourage women to deliver under skilled birth attendants or health facility should also be promoted and pregnant women need to be prepared for birth as well as for complication readiness.

## Abbreviations

AMTSL, active management of third stage of labour; HC, Health Centre; PPH, postpartum haemorrhage; TBA, traditional birth attendant; THRIVE, training health researchers into vocational excellence in East Africa; VHT, village health team
